# A mini-review of the effects of inhalational and intravenous anesthetics on oxidative stress in dogs

**DOI:** 10.3389/fvets.2022.987536

**Published:** 2022-09-12

**Authors:** Katerina Tomsič, Alenka Nemec Svete

**Affiliations:** Small Animal Clinic, Veterinary Faculty, University of Ljubljana, Ljubljana, Slovenia

**Keywords:** anesthesia, dogs, inhalational anesthetics, intravenous anesthetics, reactive oxygen species, oxidative stress

## Abstract

General anesthesia increases the production of reactive oxygen species (ROS), which can exacerbate or increase oxidative stress and thus affect the prognosis of surgical procedures. Oxidative stress has been implicated in the development of cardiovascular, dermatologic, oncologic, and other diseases in dogs, as well as ischemia and reperfusion injury. Some anesthetics, such as halogenated anesthetics, have been shown to stimulate the production of ROS, while others, such as propofol, have antioxidant properties. However, the antioxidant effects of these anesthetics may not be sufficient to counteract oxidative damage at the doses used clinically. Nevertheless, the effects of anesthetics should be considered to minimize oxidative damage during anesthesia in dogs to improve the outcome of procedures requiring general anesthesia. This mini-review addresses the current knowledge on oxidative stress during inhalational and intravenous anesthesia in dogs. There is still a lack of information on the management of anesthesia in dogs with respect to oxidative stress. Further research, including comprehensive clinical studies is needed to better understand oxidative injury mechanisms and improve perioperative protocols during anesthesia in dogs.

## Introduction

Anesthetic metabolism, changes in tissue perfusion and oxygenation during anesthesia, and surgical trauma increase the generation of reactive oxygen species (ROS), which can aggravate oxidative stress in humans (1–5) and dogs (6–9). Oxidative stress has been associated with ischemia and reperfusion injury, as well as cardiovascular, dermatologic, oncologic, and other diseases in dogs (10). In the perioperative setting, oxidative stress may be a determining factor in the outcome of surgical procedures (1, 3, 5). Therefore, the choice of an anesthetic protocol is important because some anesthetics may stimulate the production of ROS, whereas others have antioxidant properties (3, 4).

The purpose of this mini-review is to summarize the current literature on the effects of inhalational and intravenous anesthetics on oxidative stress in dogs. Relevant literature was selected using the Pubmed database and the Google Scholar search engine. The search terms were “oxidative stress anesthesia dogs.” In the section on individual anesthetics, the search terms were the anesthetic sought with the words: “oxidative stress”; “antioxidant”; “oxidative dogs” (i.e., ketamine oxidative stress; ketamine antioxidant; ketamine oxidative dogs).

## Oxidative stress, reactive oxygen species and antioxidants

The most recent definition of oxidative stress states that oxidative stress is “an imbalance between oxidants and antioxidants in favor of oxidants, resulting in disruption of redox signaling and control and/or molecular damage” (11).

Reactive oxygen species include oxygen free radicals [superoxide (${\text{O}}_{2}^{•}$), hydroxyl radical (^•^OH), peroxyl radical (ROO^•^)] and reactive nonradical species [singlet oxygen (^1^O_2_), hydrogen peroxide (H_2_O_2_), hypochlorous acid (HOCl)] (12, 13). Endogenous sources of ROS include metabolic processes in mitochondria and peroxisomes, inflammatory cellular reactions, and the catalytic action of cytochrome P450. External sources of ROS include radiation (ultraviolet, X-ray, and gamma rays), cigarette smoke, ultraviolet light, drugs, chemical reagents, industrial solvents, and other environmental pollutants (14, 15). Reactive oxygen species are involved in biological processes, such as cellular signal transduction, adaptation to stress (16), and cellular defense (17). Under pathological conditions, excessive amounts of ROS damage biologically important molecules such as lipids, proteins, and DNA and trigger reactions that can destroy the cell membrane, block the action of important enzymes, prevent normal cell division, destroy DNA, and block energy production (12, 18).

An antioxidant is any substance that delays, prevents, or eliminates oxidative damage to a target molecule (19). Antioxidants are endogenous or exogenous molecules that mitigate any form of oxidative/nitrosative stress or its consequences. The endogenous antioxidant system consists of enzymatic and non–enzymatic antioxidants. Antioxidant enzymes such as superoxide dismutase (SOD), glutathione peroxidase (GPX) and catalase (CAT) accelerate the transition from ROS to more stable products. Non-enzymatic antioxidants such as melatonin, thiol antioxidants (glutathione, thioredoxin, and lipoic acid), ubiquinone (coenzyme Q_10_), and uric acid act as free radical scavengers (12, 18, 20). Plasma albumin is a very abundant circulating antioxidant that acts either as a radical scavenger or a chelator of metal ions, heme, and other molecules (21, 22). Exogenous antioxidants supplied to the body through diet and supplements include ROS scavengers such as alpha-tocopherol (vitamin E), ascorbic acid (vitamin C), and carotenoids, as well as dietary components that enhance endogenous antioxidant activity (12, 14, 18).

The antioxidant capacity (TAC) of plasma or other body fluids represents the redox status of the organism, which is influenced by environmental and metabolic factors, physiological or pathological conditions, and dietary antioxidant intake (23–26). However, the TAC provides only a general insight into antioxidant status (27). Water-soluble antioxidants such as uric acid, ascorbic acid, proteins, and other low-molecular-weight antioxidants are retained in aqueous plasma compartments, whereas fat-soluble antioxidants (e.g., vitamin E, coenzyme Q_10_ and carotenoids) are hidden in lipoproteins (24). Determination of the antioxidant capacity of water- (ACW) and lipid-soluble (ACL) antioxidants is an accurate method for assessing the antioxidant capacity of plasma (28, 29).

Studies on the effects of anesthesia on oxidative stress in humans (3, 4, 30–33) and dogs (9, 34–46) are based on the determination of changes in antioxidant (and oxidative) capacity, activities of antioxidant enzymes, and markers of oxidative damage to DNA, proteins, and lipids.

Studies on oxidative stress during anesthesia in dogs are summarized in Table 1. A brief presentation of some of the oxidative stress parameters mentioned in the review is summarized in Table 2.

**Table 1:** Clinical studies and results on oxidative stress during anesthesia in dogs.

**Anesthetics**	**Animals**	**Surgical** **procedure**	**Parameters of oxidative stress**	**Significant findings**	**Reference**
G1 (control) - thiopentone G2 - thiopentone and hypoxia G3 - halothane G4 - halothane and hypoxia	24 mongrel dogs	Liver biopsy (experimental)	Plasma and liver vitamin E, GSH, MDA. Vitamin C and SOD in liver biopsies.	In G2, G3, G4 time dependent depletion of vitamin E and reduced glutathione in liver and plasma, and vitamin C in liver. Increased plasma and liver MDA. Hypoxia inhibited liver SOD activity.	(34)
Enflurane	10 kangal dogs	/	Serum vitamin E, A, beta carotene, GPX, MDA.	Decreased vitamin E and beta carotene, increased vitamin A and MDA.	(35)
G1 - halothane (2.5–3 vol %) G2 - halothane (0.5 vol %) + fentanyl, pancuronium bromide and controlled ventilation G3 - lumbosacral epidural with lidocaine G4 (control) - only blood sampling	24 mixed breed dogs	/	Plasma MDA	Increased blood MDA concentrations in G1 compared to baseline and other groups.	(36)
G1 - induction with midazolam and thiopental G2 - induction with midazolam and ketamine Desflurane	16 crossbreed dogs	/	Blood CAT and SOD	Time dependent increase in SOD in G1 and increase in CAT in G2.	(37)
G1 and G2 Induction with thiopental G1 - halothane G2 - isoflurane	14 mongrel dogs	Various procedures under anesthesia	Plasma MDA	None.	(38)
G1 - intramuscular anesthesia with medetomidine-tiletamine/zolazepam combination G2 - volatile anesthesia with isoflurane (2 vol % isoflurane in 100% oxygen).	10 beagle dogs	/	Plasma SOD, CAT, GPX	Decreased SOD, CAT and GPX compared to baseline in G1 and G2. Decreased CAT and GPX in G1 compared to G2.	(39)
G1 - thiopental induction G2 - propofol induction Isoflurane anesthesia	18 mongrel dogs	Laparotomy and gastrotomy	Plasma TOS, TAC, OSI	Increased TOS and OSI in G1 compared to baseline. Decreased TAC levels in G2 compared to baseline. TOS and OSI change ratio significantly lower in G2 compared to G1.	(40)
G1 - isoflurane G2 - propofol + isoflurane G3 - propofol TIVA	9 beagle dogs	/	Blood GPX, SOD, CAT	Decreased SOD and CAT activities in G1 and G2 compared to baseline. Increased CAT activity in G3 compared to baseline and to G1.	(41)
Isoflurane induction (4 vol %) G1 - 1 x MAC isoflurane G2 - 2 x MAC isoflurane	12 beagle dogs	/	Plasma TOS, TAC, OSI	Dose and time—dependent increase in TOS and OSI and decrease in TAC in G1 and G2 compared to baseline. TOS higher in G2 compared to G1; TAC and OSI higher in G2 compared to G1.	(42)
G1 - Propofol—mech vent G2 - Propofol—spont vent G3 - Isoflurane—mech vent G4 - Isoflurane—spont vent	20 mixed breed dogs	Pneumoperitoneum 15 mm Hg, 40 min	Serum TAC, MDA	Increased MDA in propofol groups. compared to isoflurane groups.	(43)
G1 - propofol G2 - sevoflurane	16 client owned dogs MMVD ACVIM B2	Periodontal treatment	Plasma Vitamin E, MDA, blood SOD, GPX	Decrease in vitamin E compared to baseline in G1 and G2. Increased GPX activity in G2 60 min after induction compared to baseline.	(9)
G1 - propofol G2 - sevoflurane G3 - healthy control, sevoflurane	30 client owned dogs MMVD ACVIM B2	Periodontal treatment	Plasma ACL, ACW	ACL elevated in G2 compared to G3 at all sampling points. Increased ACL at 60 min after induction in G1 compared to baseline. Increased ACW at 60 min in all groups, and in G1 at 6 h after induction compared to baseline. Increased ACW in G1 compared to G3 at 60 min after induction.	(44)
G1 - ketamine induction G2 - propofol induction Isoflurane anesthesia	24 client owned dogs	Osteosynthesis or Soft tissue surgery	Serum MDA, SOD, CAT, GPX	MDA decreased 30 min after induction in G1.	(46)

ACL, antioxidant capacity of lipid-soluble antioxidants; ACVIM, American College of Veterinary Internal Medicine; ACW, antioxidant capacity of water-soluble antioxidants; CAT, catalase; G, group; GPX, glutathione peroxidase; GSH, reduced glutathione; MAC, minimal alveolar concentration; MDA, Malondialdehyde; MMVD, myxomatous mitral valve degeneration; mech vent, mechanical ventilation; OSI, oxidative stress index; SOD, superoxide dismutase; spont vent, spontaneous ventilation; TOS, total oxidant status; TAC, total antioxidant capacity; 8-OHdG, 8-hydroxydeoxyguanosine.

**Table 2:** Brief presentation of the oxidative stress parameters mentioned in the review.

**Oxidative stress parameter**	**Abbreviation**	**Brief description**	**References**
Antioxidant capacity of lipid-soluble antioxidants	ACL	The plasma antioxidant capacity of lipid-soluble antioxidants includes exogenous and endogenous lipophilic antioxidants hidden in lipoproteins, such as vitamin E, coenzyme Q_10_ and carotenoids.	(29, 44)
Antioxidant capacity of water-soluble antioxidants	ACW	The plasma antioxidant capacity of water-soluble antioxidants includes antioxidants present in aqueous plasma compartments, such as vitamin C, uric acid, glutathione, proteins, and other low molecular weight antioxidants.	(28, 44)
Catalase	CAT	A major intracellular antioxidant enzyme that reduces hydrogen peroxide to water and molecular oxygen.	(37, 39, 41, 46)
7,8-dihydro-8-oxoguanine	8-oxo-Gua	A marker of oxidative DNA damage.	(33)
Free sulfhydryl groups	-SH	-SH groups are important antioxidants, scavengers of peroxides that help to protect cells from oxidative damage.	(31)
Glutathione peroxidase	GPX	A major intracellular antioxidant enzyme that catalyzes the reduction of hydrogen peroxide and lipid peroxides to water and lipid alcohols.	(9, 32, 35, 39, 41, 46, 47)
Glutathione—reduced form	GSH	A main endogenously synthesized water-soluble antioxidant.	(34)
Lipid hydroperoxide	LOOH	A marker of oxidative damage to lipids (lipid peroxidation marker).	(31)
Malondialdehyde	MDA	A marker of oxidative damage to lipids (lipid peroxidation marker); one of the most investigated secondary products of lipid peroxidation.	(9, 34–36, 38, 43, 46)
Oxidative stress index	OSI	An indicator of the degree of oxidative stress calculated as the ratio of the TOS to the TAC.	(31, 32, 40, 42)
Superoxide dismutase	SOD	A major intracellular antioxidant enzyme that catalyzes the dismutation of highly reactive superoxide radical to less reactive hydrogen peroxide that can in turn be destroyed by GPX or CAT reactions.	(9, 34, 37, 39, 41, 46, 47)
Total antioxidant capacity	TAC	TAC is the measure of the amount of free radicals scavenged by a test solution being used to evaluate TAC of a biological sample. The measure of TAC considers the cumulative action of all the antioxidants present in plasma and body fluids, thus providing an integrated parameter rather than the simple sum of measurable antioxidants. The capacity of known and unknown antioxidants and their synergistic interaction is therefore assessed, thus giving an insight into the delicate balance *in vivo* between oxidants and antioxidants. Several methods have been developed to measure the TAC of different biological samples; however, the methods vary greatly, which can result in incomparable results among studies.	(23–25, 31, 32, 40, 42, 43, 47)
Total antioxidant performance	TAP	The TAP is the measure of the TAC of plasma antioxidants localized in both aqueous and lipid compartments, including their cooperation/synergistic interaction by the use of fluorescence assay that measure the oxidizability of the lipid compartment of unfractionated plasma and is affected by lipid antioxidants as well as by hydrophilic antioxidants acting through a synergistic/cooperative mechanism.	(33)
Thiobarbituric acid reactive substances	TBARS	A marker of oxidative damage to lipids (lipid peroxidation marker).	(47)
Total oxidant status	TOS	The measurement of TOS indicates the measurement of cumulative contribution of various oxidant species in plasma.	(31, 32, 40, 42)

## Intravenous anesthetics

### Thiopental

Thiopental is a barbiturate which exert antioxidant properties *in vitro*, either by scavenging ROS (48, 49) or by inhibiting the oxidative function of neutrophils (50, 51). However, these properties have been shown to be poor compared with propofol *in vitro* (49, 52) and in human (53) and animal (3, 40, 54) studies. Lee (40) compared the effect of propofol and thiopental at induction doses on oxidative stress parameters in dogs anesthetized with isoflurane during laparotomy and gastrotomy. Oxidative stress was assessed by total plasma oxidation status (TOS), TAC and oxidative stress index (OSI). The change ratio of each value was calculated and compared between the two groups. The TOS and OSI change ratio was lower in the propofol group. Propofol was more effective in maintaining TAC compared with thiopental at induction doses, although there was a time-dependent increase in TOS and OSI and a decrease in TAC during isoflurane anesthesia in both groups (40).

### Ketamine

Ketamine, a non–competitive antagonist of N-methyl-D-aspartate receptors, has been shown to promote the formation of ROS in rats (5, 55, 56). However, according to other studies, ketamine inhibits the oxidative burst of canine peripheral blood phagocytes *in vitro* (57), and exhibits neuroprotective effects by its anti-inflammatory, antioxidant, and anti-apoptotic effects in animal models of cognitive disfunction (58). These properties were partially confirmed in a study on the effects of ketamine and propofol on cytokines, oxidative status and neutrophil functions in dogs (46). In a study on Wistar rats ketamine showed a higher antioxidant potential compared to etomidate (59). In human patients after the administration of ketamine and propofol at induction doses, the extent of lipid peroxidation (assessed by measurement of thiobarbituric acid reactive substances (TBARS) concentration) and the activity of GPX and SOD were lower in the propofol group, and plasma TAC was higher than in the ketamine group (47). Altug et al. (37) evaluated oxidative stress during desflurane anesthesia after induction with a combination of thiopental and midazolam or ketamine and midazolam. Thiopental increased SOD activity and acted as a better free radical scavenger by reducing high oxidation states of hemoglobin, whereas ketamine increased CAT activity and hemoglobin concentration. In conclusion, the authors emphasized the importance of induction agents for the antioxidant effect of desflurane anesthesia (37).

### Propofol

Propofol, a short-acting hypnotic, is an alkylphenol (2,6-diisopropylphenol) that, like vitamin E, contains a phenolic hydroxyl group (OH). It reacts with free radicals to form a phenoxyl radical (60, 61). Like vitamin E, propofol acts synergistically with ascorbic acid, a water-soluble antioxidant that converts the propofol radical to the phenolic form (62). Due to its lipophilic nature, propofol may increase fluidity and strengthen the cell membrane against physical and hemodynamic stressors (62–64). In addition, propofol could substitute for vitamin E, especially when vitamin E stores are acutely depleted and need to be replaced rapidly (65, 66). The protective antioxidant effect of propofol has been demonstrated in experimental models of brain, liver, and heart injury (52, 67–71), isolated microsomes (65), neuroblastoma cell lines (72), and human clinical trials (47, 52, 73–77). It is manifested either by reaction with peroxyl radicals and the formation of less harmful phenoxyl radicals or by reaction with peroxynitrite (78, 79). Acquaviva et al. (80) demonstrated the neuroprotective effect of propofol on astroglial cells *in vitro* through its anti-apoptotic effect, reduction of cytotoxicity, and prevention of DNA damage by peroxynitrite (80).

Braz et al. (33) investigated the effects of anesthesia maintained with isoflurane or propofol on antioxidant status in healthy adults undergoing minor surgery. The study design assessed the antioxidant capacity of the aqueous plasma compartment (hydrophilic antioxidant capacity), total antioxidant performance (TAP) and individual antioxidants. Lipid-soluble antioxidants were represented by carotenoids, retinol (vitamin A), tocopherols, lycopenes, and others, whereas uric acid represented the hydrophilic antioxidants. The oxidized purine form, 7,8-dihydro-8-oxoguanine (8-oxo-Gua), in lymphocytes was examined to assess genetic oxidative damage. The results showed a decrease in alpha-tocopherol in both groups, whereas there was an increase in gamma-tocopherol in the propofol group compared with baseline values and compared with the isoflurane group after 2 h of anesthesia. The hydrophilic antioxidant capacity and TAP were increased in both groups compared with baseline, with no differences between the two groups. The antioxidant effect was dose dependent. The authors concluded that both anesthetic regimens increased hydrophilic antioxidant capacity and TAP and did not cause oxidative DNA damage (33).

Lee and Kim (41) demonstrated the effect of propofol at induction doses and as total intravenous anesthesia (TIVA) on the activity of SOD, GPX, and CAT in dogs. The three treatments were: Group 1, 2% isoflurane; Group 2, anesthesia induced with propofol and maintained with 1.5–2% isoflurane; Group 3, TIVA with propofol. Anesthesia was maintained for 60 min. The activity of SOD decreased from baseline to the end of anesthesia in the isoflurane groups. Catalase activity decreased from baseline to the end of anesthesia and 24 h after anesthesia in the isoflurane groups. In the propofol-TIVA group, CAT activity increased at the end and 24 h after anesthesia and was higher than in the group of dogs in which isoflurane was used to induce and maintain anesthesia (41).

Tomsič et al. (9, 44) examined the effects of propofol on oxidative stress in dogs with early-stage myxomatous mitral valve degeneration (MMVD) undergoing periodontal treatment. In one study, the authors compared the effects of TIVA with propofol to anesthesia induced with propofol and maintained with sevoflurane on vitamin E, SOD, GPX, and lipid peroxidation marker malondialdehyde (MDA) (9). The authors found no significant differences between the two anesthetic protocols for any of the oxidative status parameters measured. Compared with baseline values, vitamin E concentration decreased during anesthesia in both groups, and GPX activity increased 60 min after induction of anesthesia in the sevoflurane group. In another study, the authors examined the effect of TIVA with propofol and sevoflurane anesthesia after induction with propofol on plasma ACL and ACW levels in dogs with early-stage MMVD. Dogs without signs of MMVD (control group) were induced to anesthesia with propofol and maintained with sevoflurane. Anesthesia increased ACW values in all groups, although they were higher than baseline only in the propofol group after anesthesia. Additionally, 60 min after induction to anesthesia, ACW was higher in the MMVD/Propofol group compared to the MMVD/Propofol + Sevoflurane group. Furthermore, only propofol anesthesia increased ACL levels in dogs with MMVD compared with basal levels. This could be attributed to the antioxidant properties of propofol. The authors concluded that propofol may be more suitable than sevoflurane for anesthesia of dogs with early-stage MMVD in terms of antioxidant capacity (44).

Alipour et al. (43) investigated the effects of anesthesia with propofol and isoflurane on endocrine and oxidative variables during pneumoperitoneum in dogs. The authors found that TIVA with propofol, either with or without mechanical ventilation, can increase MDA production at the end of pneumoperitoneum, whereas none of the anesthetic techniques affected thyroid and cortisol levels (43).

## Inhalational anesthetics

Inhalational anesthetics include the halogenated ethers (isoflurane, sevoflurane, desflurane, and enflurane), the alkane halothane, and the inorganic gaseous anesthetics (nitrous oxide and xenon) (3). Sevoflurane and isoflurane are the most used volatile anesthetics in veterinary practice.

Halogenated anesthetics trigger the phenomenon of ischemic preconditioning (81), an adaptive response to brief episodes of ischemia that allows protection of the myocardium from subsequent life-threatening ischemia and acts through the cellular signaling pathway. The volatile anesthetics cause the formation of ROS, initiating the signaling cascade that reduces the production of ROS in mitochondria during ischemia. Reactive oxygen species activate mitochondrial potassium-dependent ATP channels, presumably by activating a protein kinase C isoform. This results in less depolarization of the mitochondrial membrane potential and a lower electrochemical gradient for calcium ions. The reduced accumulation of calcium ions in mitochondria and the opening of mitochondrial pores protect mitochondria from damage (81). In addition, the bioavailability of ATP increases and the formation of ROS in the ischemic phase decreases (82).

### Isoflurane

Isoflurane is a commonly used halogenated ether with a low metabolization rate and solubility (83). The effects of isoflurane on oxidative stress have not been fully elucidated and remain controversial. In an experimental model using human neuroglioma cells and mouse brain tissue, Ni et al. (84) sought to elucidate the mechanisms of oxidative damage to DNA by isoflurane. The phosphorylated form of histone protein H2A variant X at Ser139 (γH2A.X) was selected as a marker of oxidative DNA damage. The results of the study showed that isoflurane induced DNA damage at clinically relevant concentrations as determined by the increase in γH2A.X in human neuroglioma cells (84). In contrast, in the clinical study by Braz et al. (33) isoflurane anesthesia did not induce DNA damage assessed by determination of 8-oxo-Gua in healthy adults (33).

In dogs, isoflurane increases oxidative stress in a dose- and time-dependent manner, as shown by Lee (42). Beagle dogs were anesthetized with different minimum alveolar concentrations (MAC) of isoflurane. Dogs in group 1 received 1.28% (1 x MAC) isoflurane, whereas dogs in group 2 received 2 × MAC. The oxidant and antioxidant status of the dogs was determined by TOS, TAC, and OSI. The levels of TOS and OSI increased significantly, while the levels of TAC decreased in both groups after anesthesia. Changes were observed in group 1 60 min, and in group 2 30 and 60 min after induction of anesthesia. The levels of TOS were higher in group 2 than in group 1 at 30 and 60 min after induction of anesthesia, while TAC and OSI were significantly higher in group 2 than in group 1 at 60 min after induction to anesthesia (42). Yarsan et al. (38) compared the effects of halothane and isoflurane on plasma MDA concentrations in dogs. Changes were not significant; however, the MDA concentration was lower under halothane anesthesia, and levels returned to baseline within 24 hours (38).

### Sevoflurane

Sevoflurane is metabolized in the liver by the isoform of the cytochrome P450 enzyme CYP2E1 (85, 86), and its metabolism has been shown to accelerate the formation of ROS (85) and impair energy metabolism in mitochondria (87, 88). Yalcin et al. (31) investigated the effects of desflurane and sevoflurane on selected oxidative stress parameters [TOS, TAC, lipid hydroperoxide (LOOH), total free sulfhydryl groups (-SH), and OSI] in mothers and neonates after elective cesarean section. Compared with baseline values, TOS and OSI were decreased in both groups. However, LOOH, TOS, and OSI were higher in maternal serum and umbilical artery blood in the desflurane group compared with the sevoflurane group. In addition, LOOH and -SH values were lower in the sevoflurane group compared with preoperative values, and there was no difference between groups in umbilical artery -SH and TAC values. The authors concluded that anesthetics could alter oxidative stress indices, and sevoflurane showed more favorable effects compared with desflurane (31). Similarly, Erbas et al. (32) compared the effects of sevoflurane, desflurane, and propofol on the oxidant and antioxidant systems of patients undergoing laparoscopic cholecystectomy. The oxidative stress parameters selected were TOS, TAC, and GPX. Compared with preoperative levels, there was an increase in postoperative TAC levels in the propofol and sevoflurane groups, and in postoperative TOS levels in the desflurane group. Glutathione peroxidase activity remained unchanged in both groups (32). These results are in general agreement with clinical studies investigating oxidative stress in dogs with early-stage MMVD during propofol and sevoflurane anesthesia (9, 44).

### Other halogenated anesthetics

Naziroglu and Günay (35) investigated the effect of enflurane on serum concentrations of vitamins A, E, beta-carotene, GPX, lipid peroxidation, and biochemical and hematological parameters in healthy dogs. The results showed a decrease in serum vitamin E and beta-carotene concentrations, while serum MDA and vitamin A concentrations were increased during enflurane anesthesia. The authors pointed out that administration of antioxidant compounds such as vitamin C, vitamin E, and selenium may be beneficial in anesthetic complications (35).

El-Bassiouni et al. (34) studied the involvement of ROS and antioxidant defense mechanisms in liver tissue and plasma under different hypoxic conditions during halothane anesthesia. In liver tissue and plasma, there was an increase in MDA and a decrease in the free radical scavengers reduced glutathione (GSH), ascorbic acid, and especially vitamin E, with hypoxia being a major contributing factor. In addition, hypoxia and halothane inhibited hepatic SOD activity (34). Simeonova et al. (36) compared the effects of three anesthetic protocols on lipid peroxidation in dogs. Halothane anesthesia increased plasma MDA concentrations in dogs compared with the fentanyl and halothane groups and with dogs treated with lumbosacral epidural anesthesia (36).

## Discussion

Intravenous and inhalational anesthetics can cause dose- and time-dependent cardiovascular and respiratory depression (59, 60) that may lead to hypoperfusion and hypoxia. In contrast, ketamine increases myocardial work and cardiac output, maintaining arterial pressure and heart rate (59). It has a mild effect on the respiratory system but can cause respiratory depression when used with other central nervous depressants (46). Anesthetic procedures in clinical trials include various agents for sedation, gentle induction of anesthesia, and analgesia. The metabolism of these agents and the stress response to pain during surgical procedures may aggravate the oxidative status of animals under anesthesia (7). The variability of anesthetic protocols and procedures may explain the conflicting results of the studies included in the present review. The preanalytical procedures and the wide variability in the methods used to measure oxidative stress parameters render some of the results incomparable between studies and may also be the reason for the conflicting results.

## Conclusion

The effect of general anesthetics on oxidative stress is variable and not yet fully understood. Comprehensive studies are needed to investigate the effect of an anesthetic on oxidative status in dogs. These studies should include larger numbers of animals and measurement of a broader range of oxidative status parameters, including markers of oxidative damage to all biologically important molecules (lipids, proteins, and DNA), concentrations of antioxidants and activity of antioxidant enzymes, and measurement of the oxidative and/or reductive potency of a biological fluid.

## Author contributions

KT and ANS conceptualized the manuscript. KT conducted the literature search and review and drafted the manuscript. ANS reviewed and improved the manuscript. Both authors approved the final version of the manuscript.

## Funding

The publication of this review was funded by the Slovenian Research Agency, Grant No. P4-0053. The funder was not involved in the manuscript preparation.

## Conflict of interest

The authors declare that the research was conducted in the absence of any commercial or financial relationships that could be construed as a potential conflict of interest.

## Publisher's note

All claims expressed in this article are solely those of the authors and do not necessarily represent those of their affiliated organizations, or those of the publisher, the editors and the reviewers. Any product that may be evaluated in this article, or claim that may be made by its manufacturer, is not guaranteed or endorsed by the publisher.
